# Identification of *Rhipicephalus microplus* Genes That Modulate the Infection Rate of the Rickettsia *Anaplasma marginale*

**DOI:** 10.1371/journal.pone.0091062

**Published:** 2014-03-07

**Authors:** Ricardo F. Mercado-Curiel, María L. Ávila-Ramírez, Guy H. Palmer, Kelly A. Brayton

**Affiliations:** Program in Vector-Borne Diseases, Department of Veterinary Microbiology and Pathology and Paul G. Allen School for Global Animal Health, Washington State University, Pullman, Washington, United States of America; Kansas State University, United States of America

## Abstract

Arthropod vectors transmit a diversity of animal and human pathogens, ranging from RNA viruses to protozoal parasites. Chemotherapeutic control of pathogens has classically focused either on insecticides that kill the vector itself or antimicrobials for infected patients. The limitation of the former is that it targets both infected and uninfected vectors and selects for resistant populations while the latter requires prompt and accurate diagnosis. An alternative strategy is to target vector molecules that permit the pathogen to establish itself, replicate, and/or develop within the vector. Using the rickettsial pathogen *Anaplasma marginale* and its tropical tick vector, *Rhipicephalus microplus*, as a model, we tested whether silencing specific gene targets would affect tick infection rates (the % of fed ticks that are infected with the pathogen) and pathogen levels within infected ticks. Silencing of three *R. microplus* genes, CK187220, CV437619 and TC18492, significantly decreased the *A. marginale* infection rate in salivary glands, whereas gene silencing of TC22382, TC17129 and TC16059 significantly increased the infection rate in salivary glands. However in all cases of significant difference in the infection rate, the pathogen levels in the ticks that did become infected, were not significantly different. These results are consistent with the targeted genes affecting the pathogen at early steps in infection of the vector rather than in replication efficiency. Identifying vector genes and subsequent determination of the encoded functions are initial steps in discovery of new targets for inhibiting pathogen development and subsequent transmission.

## Introduction

Arthropod vectors transmit a diversity of animal and human pathogens, ranging from RNA viruses to protozoal parasites. Chemotherapeutic control of pathogens has classically focused either on insecticides that kill the vector itself or antimicrobials for infected patients. The limitation of the former is that it targets both infected and uninfected vectors and thus broadly selects for resistant populations while the latter requires prompt and accurate diagnosis. An alternative strategy is to target vector molecules that permit the pathogen to establish itself, replicate, and/or develop within the vector, thus specifically targeting only the small proportion of infected vectors.

Vector competence, the ability to acquire and transmit pathogens, is a multifactorial process and involves multiple genes and gene networks in multiple organs. The vector midgut and salivary glands are attractive targets as these organs represent, respectively, sites of initial colonization and secretion into the saliva for transmission [1], [2], [3], [4], [5]. Using the rickettsial pathogen *Anaplasma marginale* and its tropical tick vector, *Rhipicephalus microplus*, as a model, we previously identified a set of tick midgut and salivary gland genes that are regulated in response to pathogen infection [5]. We supplemented this set with *R. microplus* genes for which the expressed protein has been shown to vary in response to babesial infection [6], [7]. Six candidate genes were selected based on bioinformatics analysis and an initial screen using post-transcriptional gene silencing by small interfering RNA (siRNA) (Table 1). Silencing of these six genes was then used to test two related hypotheses in the *A. marginale*/*R. microplus* model. The first was that silencing of the selected *R. microplus* genes affects the *A. marginale* infection rate (the % of fed ticks that acquire infection) in the tick midguts or salivary glands. The second hypothesis was that silencing of the selected *R. microplus* genes affects the level of *A. marginale* within infected ticks. Herein, we present the results of these experiments and discuss the findings in the context of the interface between tick biology and pathogen transmission.

**Table 1:** Bioinformatic analysis of candidate genes.

	Annotation (putative)	Best alignment^a^	Species^b^	E value	5′ end^c^	TM^d^	SP^e^	CDD
TC18492	Secreted protein	XP_002435215	Is	3e-67	N	Y	Y	–
TC16059	aldehyde dehydrogenase	XP_002412591	Is	0	N	Y	N	CD07141
TC17129	glutamine synthetase	XP_967731	Tc	5e-133	N	N	N	PLN03036
TC22382	NADH-ubiquinone reductase	DAA34117	Av	6e-146	N	N	N	pfam10588 pfam13510
CK187220	unknown	–	–	–	?	N	N	-
CV437619	Tat binding protein 1-interacting protein	XP_002409139	Is	1e-86	Y	N	N	PHA02592 CD07599

a Reports the GenBank accession number of the sequence with the lowest e value.

b Reports the species with the most similar homolog; Is = *I. scapularis*, Tc = *Tribolium castaneum*, Av = *Amblyomma variegatum*.

c Indicates whether the alignment with a known protein indicates the presence of the first coding amino acid in the cDNA sequence; Y = yes; N = no.

d Indicates whether there are any recognized transmembrane domains using TMpred; Y = yes; N = no.

e Indicates the presence of a signal peptide; Y = yes; N = no. Note: if No is indicated in the 5′ end column, the positive SP prediction may actually reflect the presence of a TM domain near the start of the sequence rather than a true signal peptide.

## Materials and Methods

### Experimental Animals and Ticks

Animals were maintained according to IACUC protocol #2010-54 authorized by the University of Idaho Institutional Animal Care and Use Committee in accordance with institutional guidelines based on the U.S. National Institutes of Health (NIH) Guide for the Care and Use of Laboratory Animals. Two Holstein calves, 4 months of age (#C36185, #C36190), were used in this study. These animals had no previous exposure to ticks. One animal was inoculated intravenously with approximately 10^9^ *A. marginale* (St. Maries strain). The second uninfected calf was used for rearing 4 grams, approximately 80,000 larvae, of *Rhipicephalus microplus* ticks (La Minita stock) to the engorged nymph stage. Molting nymphs were manually collected from the calf after 14 days, and incubated at 26°C, 95% humidity to complete molting to the adult stage. Unfed adult ticks were sorted by sex and the males used for silencing of selected genes within 36 hrs of molting.

### Small Interfering RNA

Two different double-stranded siRNAs were specifically designed and chemically synthesized for each selected gene (Integrated DNA Technologies, Inc). Synthetic short RNA duplexes had a 2-base 3′-overhang on the antisense strand, and were blunt on the other end; the 3′ end of the sense strand contained two DNA instead of RNA bases. The two siRNA duplexes designed for each selected gene are listed in Table 2. The siRNAs were suspended in Nuclease Free Duplex Buffer (Integrated DNA Technologies, Inc).

**Table 2:** Sets of siRNAs, primers and probes used for each target gene.

Gene	Set	siRNA sequence (5′ - 3′)^a^	Primer/Probe sequences (5′ - 3′)^b^
CK187220	A	A: UCU GUG AGC UUA UAG UGG AUU GUG GAG	F: GCT TCC TGA ATT GCA TTA AGC TCC G
		S: CCA CAA UCC ACU AUA AGC UCA CAG A	R: AAC ATG CAA CAT GTC GGC TTG AGG
	B	A: AUU GAA UUU CGG AGC UUA AUG CAA UUC	P: AGA GGT CCC TCA TCT TGG CAA GTT GT
		S: AUU GCA UUA AGC UCC GAA AUU CAA T	
CV437619	A	A: UUU CCG UAG GUC UUC UCU UUG AUC UUU	F: AGC GCT TGA GTA TGT GCT TTG TGG
		S: AGA UCA AAG AGA AGA CCU ACG GAA A	R: CAA CGC GAA ACT GAC CGA AAC GTA
	B	A: AGG UUG UUG AAG AUG UCG UUG GAG CUG	P: AGT AGT GTC TGC ACG TGC ACG CAA AT
		S: GCU CCA ACG ACA UCU UCA ACA ACC T	
TC18492	A	A: CUC UUC ACA CUC ACC UUG AUU UCU CCG	F: AAC TTC ACC ACC ACA TTC ACT GGC
		S: GAG AAA UCA AGG UGA GUG UGA AGA G	R: TGT CGG TAT CTG TTC TGT CTT CGG
	B	A: GUG CUG UUA CGG UCG UAC UUG AGC UGG	P: AAA CTC CCA AAC TCA GAA GCT GCG CT
		S: AGC UCA AGU ACG ACC GUA ACA GCA C	
TC22382	A	A: GGA UGG UUC AUC AGC AAG AAC UCC AUG ACU CCC	F: AAC AAC AGA AAC AGC AGG CGA AGC
		S: GAG UCA UGG AGU UCU UGC UGA UGA ACC AUC C	R: CCA CGG GCT TTG GAG ATT TCT CTA CT
	B	A: CCU CGC UCA AGC UGU CGU AAG GCA GAG GCA UCC	P: AAA TCC CGC GTT TCT GCT ACC ACG A
		S: AUG CCU CUG CCU UAC GAC AGC UUG AGC GAG G	
TC17129	A	A: UGU CAA UUC AAC AGC AAU GAG UAG CUU	F: TGA CAT CAC GCC ACT CGA GAT TGT
		S: GCU ACU CAU UGC UGU UGA AUU GAC A	R: TGT GAG TGC CAC CAC GAG ATA CAT
	B	A: CAU GAA UGA UAU ACC AUC CCA CUG UUU	P: TCA TTC ATG CCC ATG GTG CAG TGT
		S: ACA GUG GGA UGG UAU AUC AUU CAT G	
TC16059	A	A: AUC GUC AAU CUG UGG UCC UUG UUC GGU	F: AGG ACC ACA GAT TGA CGA TGA GCA
		S: CGA ACA AGG ACC ACA GAU UGA CGA T	R: TGA ACT TGA GGA TCT GCT GGA CTG
	B	A: GGG AUC UUG AUU GUG ACC GUC UUU GUU	P: AAG CGA ATT GGC AAC GAG GGC TAC TT
		S: CAA AGA CGG UCA CAA UCA AGA UCC C	
*R. microplus* actin			F: AAG CGT GGT ATC CTC ACC CTG AAG TA R: AGG TCT CGA ACA TGA TCT GCG TCA
*msp5*			F: CTT CCG AAG TTG TAA GTG AGG GCA
			R: CTT ATC GGC ATG GTC GCC TAG TTT
			P: GCC TCC GCG TCT TTC AAC AAT TTG GT

a A = Antisense; S = Sense strand.

b F = Forward, R = Reverse, P = Probe.

### Labeling and Injection of Ticks with siRNA

Freshly molted male ticks were allocated to specific treatment groups and ticks within a group identified by removal of a single leg between the third and fourth segment. Ticks were injected with 0.5 µl of a 10 pmol/µl stock solution of one of the specific siRNA duplexes described above. Control groups were injected with an equivalent volume of Nuclease Free Duplex Buffer (Integrated DNA Technologies, Inc). The injection was performed using a 10 µl syringe (Hamilton) with a borosilicate glass needle coupled to a 33 gauge 15 mm metal needle (Hamilton), and the desired administered volume was controlled by the UMP3 Microsyringe Injector and Micro4 Controller (World Precision Instruments). The glass needles were made from borosilicate glass capillaries (Harvard Apparatus) using a P2000 laser-based micropipette puller (Sutter Instrument Co.). The injection procedure was carried out at the base of the 4^th^ left leg through the sclerotized coxal membrane. No reflux of the injected solution, hemolymph or tissue was observed from the site of the puncture when the glass needle was carefully withdrawn.

Approximately 5 hrs following the corresponding procedure, labeled/injected tick groups were allowed to acquisition feed on the *A. marginale* infected calf during acute bacteremia. Ticks were allowed to feed for 6 days and then removed and individually dissected for collection of salivary glands or midguts within 48 hrs. One half of the tissue was put in Trizol (Invitrogen), and the other half in Cell Lysis Buffer (Qiagen) containing 2 mg/ml proteinase K (Invitrogen), and stored at −70°C until total RNA or genomic DNA extractions were performed for gene silencing, or infection level/rate and β-actin level determinations, respectively.

### Gene Silencing, A. marginale Infection and R. microplus Actin Determination in Single Tick Tissues

In order to assess the gene silencing effect, total RNA extracted from dissected tissues, either half of the midgut or one salivary gland, was treated with DNase (Applied Biosystems). Random primed, single stranded cDNA was synthesized using the SuperScript III First-Strand Synthesis SuperMix for qRT-PCR kit (Invitrogen), and analyzed by TaqMan quantitative PCR (qPCR), using iQ-Supermix reagents (BioRad, Hercules, CA), to determine the gene expression level of each siRNA-target gene; *R. microplus* actin was used for normalization. All Taqman qPCR assays were carried out using aliquots from the same cDNA sample. Control reactions without reverse transcriptase or cDNA were carried out to confirm the absence of DNA contamination in the RNA samples or contamination in the qPCR reaction, respectively. The sets of primers and TaqMan probes used for each analyzed gene are listed in Table 2. All assays were done in triplicate.

Genomic DNA extracted from dissected tissues, either half of the midgut or one salivary gland, was analyzed by qPCR for the single copy gene *msp5* to determine the *A. marginale* infection level and *R. microplus* actin level as previously described [5], [8]. qPCR assays were carried out in triplicate for the unknown DNA samples, simultaneously with serial dilutions of cloned *msp5* or *actin*, using the iQ-Supermix (BioRad, Hercules, CA). The primers and TaqMan probes used are listed in Table 2. The number of positive samples that showed a quantifiable amount of *A. marginale* was used to determine the infection rate, dividing it by the total number of samples in the corresponding group of singly collected tick tissues.

### Statistical Analysis

Statistical analysis of gene silencing data (gene copies/10^3^ β-actin), infection level (mean no. bacteria/total organ), and *R. microplus* actin level in individual organ (β-actin gene copies×10^5^/organ) were performed using Minitab 15 version 1.30.0; consisted of One-way analysis of variance (ANOVA) with a factor of three and six, sixteen, and sixteen levels, respectively, and Tukey’s family error rate of 5; p values of less than 0.05 were considered statistically significant.

## Results

### Selection of Genes

Using a gene expression microarray we identified several *R. microplus* genes whose expression is regulated upon infection with *A. marginale* [5]. We supplemented this set with *R. microplus* genes encoding proteins that were described as being regulated upon *Babesia bovis* infection, a protozoal pathogen that uses the same tick vector [6], [7]. We selected six genes from these sets using a combination of results from a pilot silencing experiment and bioinformatics analysis (Table 1; see http://compbio.dfci.harvard.edu/cgi-bin/tgi/gimain.pl?gudb=b_microplus for BmiGI2.1 listings of genes). TC18492 showed one of the highest fold changes in transcription in *A. marginale* infected tick salivary glands, 3.7 and 6.5 at 6 and 9 days, respectively, which was congruent with the protein overexpression in the tick upon *B. bovis* infection [5], [6]. TC16059 and TC17129 showed increased protein expression upon *B. bovis* infection, respectively, while mRNA levels remained unaffected upon *A. marginale* infection. CK187220 was transcriptionally down-regulated during early infection and, subsequently, in the salivary gland. As a control, CV437619 was selected as a control as there was no evidence of regulation upon pathogen infection in the tick salivary gland [5], [6], [7]. These five genes were examined in the salivary gland, which is the relevant tissue in which *A. marginale* undergoes final replication prior to transmission. TC22382 was examined in both the tick midgut and the salivary gland based on the two factors: i) bioinformatic analysis that suggested a possible role of this gene in electron and proton transport that may affect specific midgut physiological processes like uptake of blood meal components, diuresis and water balance (Table 1); and ii) discrepant results in the midgut following infection with the two different pathogens: increased protein expression in the midgut upon *B. bovis* infection but a 5-fold decrease in mRNA levels with *A. marginale* infection.

### Tick Survival

Because injection of adult male *B. microplus* ticks had not been previously reported, we first determined the survival rates for this procedure as well as for the procedure used to identify ticks by treatment group, removal of one of the eight legs. Tick survival was evaluated as the proportion of treated ticks that were recovered alive after 20 days of feeding (6 days after Nuclease Free Duplex Buffer injection and/or clipping of one leg for identification). Ticks subjected to both injection and clipping of a leg had a much lower survival (27%); this data was used to determine the group size to be used for injection of siRNA.

### Gene Silencing with Two Different Specific siRNAs

**Figure 1:**
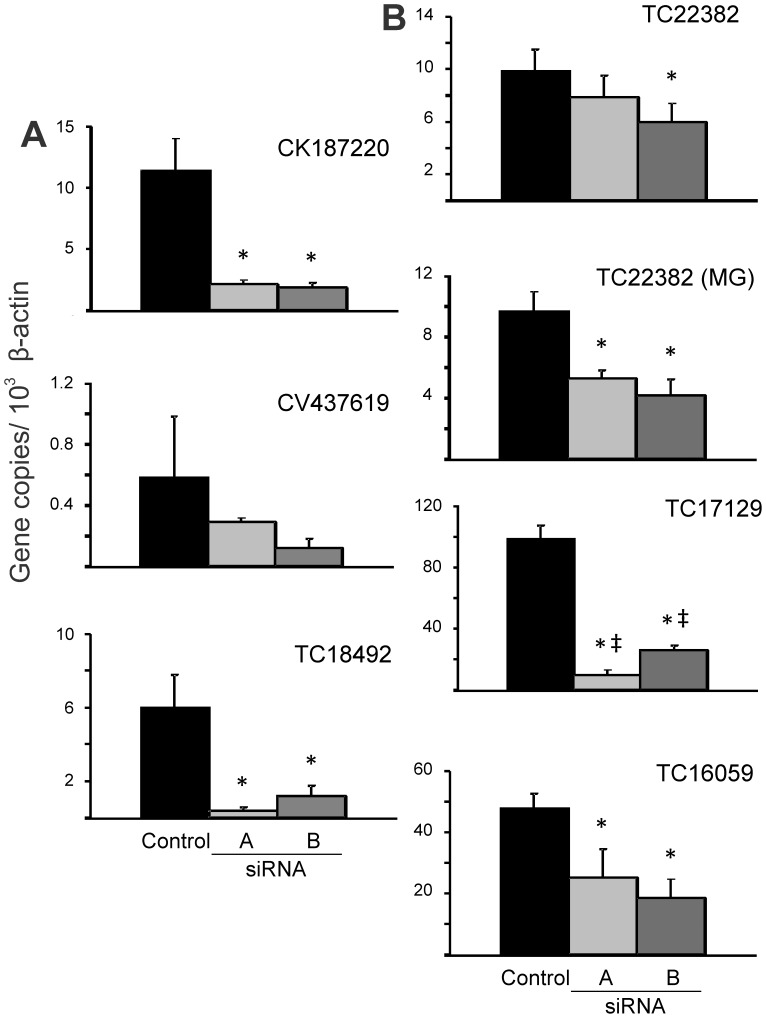
Gene silencing effect of gene-specific siRNAs. The expression of targeted genes in salivary glands or midgut (for TC22382 only) was analyzed by qRT-PCR. Data are presented as mean values of gene copies with the standard deviation indicated with error bars, normalised relative to β-actin transcript level. Two double-stranded siRNAs were designed for each gene: siRNA_A and siRNA_B. Control ticks were injected with Nuclease Free Duplex Buffer. Asterisk (*) and double dagger (‡) indicate statistically significant difference (p<0.05) when comparing to the control group and between both gene-specific siRNA groups, respectively. Panel A represents genes whose silencing resulted in a lower *A. marginale* infection rate, while panel B represents genes whose silencing resulted in a higher *A. marginale* infection rate.

Evidence for off-target effects of siRNA in arthropod systems has been reported [9], [10], [11], [12]. In an effort to control for off-target effects, two different double-stranded siRNAs were specifically designed for each gene and are referred to as (gene identifier) siRNA_A and (gene identifier) siRNA_B. The possibility of having equivalent specific and off-target effects with the use of two different siRNAs are low, and provide better support that the resulting phenotype is due to a specific inhibition of the cognate mRNA. The effect of siRNAi is systemic with gene silencing effects occurring throughout the whole tick [13], [14]. RNA extracted from individual salivary glands or from half a midgut was analyzed by qRT-PCR to determine the gene silencing effect (Figure 1).

Injection with CK187220 siRNA_A and CK187220 siRNA_B resulted in a statistically significant silencing effect of 81% and 84%, respectively, in salivary glands. There was no significant difference in the silencing effects of the two siRNAs (Figure 1). Treatment with CV437619 siRNA_A and CV437619 siRNA_B resulted in salivary gland expression levels of CV437619 that were not significantly different as compared to the controls (Figure 1). This may be due to the low expression levels of CV437619 in the controls, making it more difficult to detect a significant reduction following siRNA treatment. TC18492 siRNA_A and TC18492 siRNA_B caused a statistically significant silencing effect of 93% and 80%, respectively in salivary glands. There was no significant difference between the effects of the two siRNAs (Figure 1). The silencing effects of TC17129 siRNA_A and TC17129 siRNA_B in salivary glands were 90% and 73%, respectively, which were statistically significantly different both one from another and as compared to the control group (Figure 1). Both TC16059 siRNA_A and TC16059 siRNA_B caused a statistically significant silencing effect of 47% and 61% in salivary glands, respectively; there was no significant difference between the effects of both siRNAs (Figure 1).

The silencing effect of TC22382 siRNA_A and TC22382 siRNA_B were investigated in the midgut as well as in the salivary glands due to the potential role of this transporter to affect midgut physiology involving uptake of bloodmeal components, diuresis, and water balance. In the midgut TC22382 siRNA_A and TC22382 siRNA_B caused a statistically significant silencing effect of 45% and 57%, respectively; there was no significant difference between the effects of the two siRNAs (Figure 1). In salivary glands, the reduction was 20% and 40%, respectively (Figure 1). Only the TC22382 siRNA_B group was significantly different as compared to the control group.

### Effect of Gene Silencing on *A. marginale* Infection Rate and Level

During acquisition feeding, the ticks were exposed to *A. marginale* levels ranging from 6×10^7^–8.5×10^8^ organisms/ml of blood. Control ticks had infection rates (% of fed ticks that acquired infection) of 100% and 60% in midgut and salivary glands, respectively (Table 3). Silencing with both members of each set of gene-specific siRNAs, siRNA_A and siRNA_B, showed the same outcome in all cases. All three possible outcomes were observed with one or more of the gene-specific siRNA sets: an increase, decrease or no effect on the infection rate (Table 3). Gene silencing of CK187220, CV437619, and TC18492 resulted in statistically significant decreases in the salivary gland infection rate, whereas gene silencing of TC17129 and TC16059 significantly increased the infection rate. Silencing of TC22382 in the salivary gland also resulted in a statistically significant increase in infection rate; however no increase was detectable in the midgut as the control ticks also had a 100% infection rate (Table 3). The corresponding infection level for each group reflects the mean infection level of samples within the group that showed a quantifiable amount of *A. marginale* (Table 3). The infection levels, reported as the mean number of organisms per salivary gland pair or midgut, were not statistically significantly different when comparing both gene-specific siRNA injected groups with each other or with their respective salivary gland or midgut control groups (Table 3).

**Table 3:** *Anaplasma marginale* infection levels and rates following gene silencing.

				Infection Level	Infection Rate	Survival Rate
InfectionRate	Group	Injected With	TissueAnalyzed^a^	Mean no. bacteria/totalorgan (±SD)^b^	(No. positive/No.recovered)×100	(No.recovered/No. injected) ×100
	Control	Nuclease Free	SG	2.85×10^4 (±1.28)^	59.45 (22/37)	13.21 (37/280)
		Duplex Buffer	MG	1.00×10^4 (±0.43)^	100 (8/8)^c^	
Lower	CK187220	CK187220 siRNA_A	SG	1.07×10^4 (±0.16)^	41.66 (15/36)	20 (36/180)
	CK187220	CK187220 siRNA_B	SG	2.74×10^4 (±0.45)^	30.43 (7/23)	12.77 (23/180)
	CV437619	CV437619 siRNA_A	SG	2.12×10^4 (±2.18)^	31.25 (15/48)	26.66 (48/180)
	CV437619	CV437619 siRNA_B	SG	1.45×10^3 (±1.66)^	41.66 (10/24)	13.33 (24/180)
	TC18492	TC18492 siRNA_A	SG	5.09×10^3 (±0.54)^	40 (10/25)	12.5 (25/200)
	TC18492	TC18492 siRNA_B	SG	1.25×10^5 (±1.80)^	45.45 (15/33)	16.5 (33/200)
Higher	TC22382	TC22382 siRNA_A	SG	4.05×10^3 (±0.35)^	100 (3/3)	3 (3/100)
	TC22382	TC22382 siRNA_B	SG	8.10×10^2 (±0.54)^	100 (15/15)	15 (15/100)
	TC17129	TC17129 siRNA_A	SG	8.86×10^4 (±0.97)^	77.77 (7/9)	11.25 (9/80)
	TC17129	TC17129 siRNA_B	SG	1.41×10^4 (±1.14)^	82.60 (19/23)	28.75 (23/80)
	TC16059	TC16059 siRNA_A	SG	1.18×10^4 (±0.22)^	87.5 (7/8)	10 (8/80)
	TC16059	TC16059 siRNA_B	SG	7.49×10^4 (±0.51)^	90 (18/20)	25 (20/80)
Equal	TC22382	TC22382 siRNA_A	MG	7.90×10^4 (±0.95)^	100 (3/3)	3 (3/100)^d^
	TC22382	TC22382 siRNA_B	MG	1.53×10^4 (±0.63)^	100 (15/15)	15 (15/100)^d^
Mean						15.83 (304/1920)

a SG = Salivary glands, MG = midguts.

b SD = Standard deviation.

c 8 ticks from the salivary gland control group were also analyzed in the midgut group.

d These values were not considered when calculating the mean rate since these midguts where dissected from the same ticks that were used for dissecting salivary glands for TC22382 siRNA_A and TC22382 siRNA_B analysis.

### Effect of Gene Silencing on Tissue Development/Maintenance

**Figure 2:**
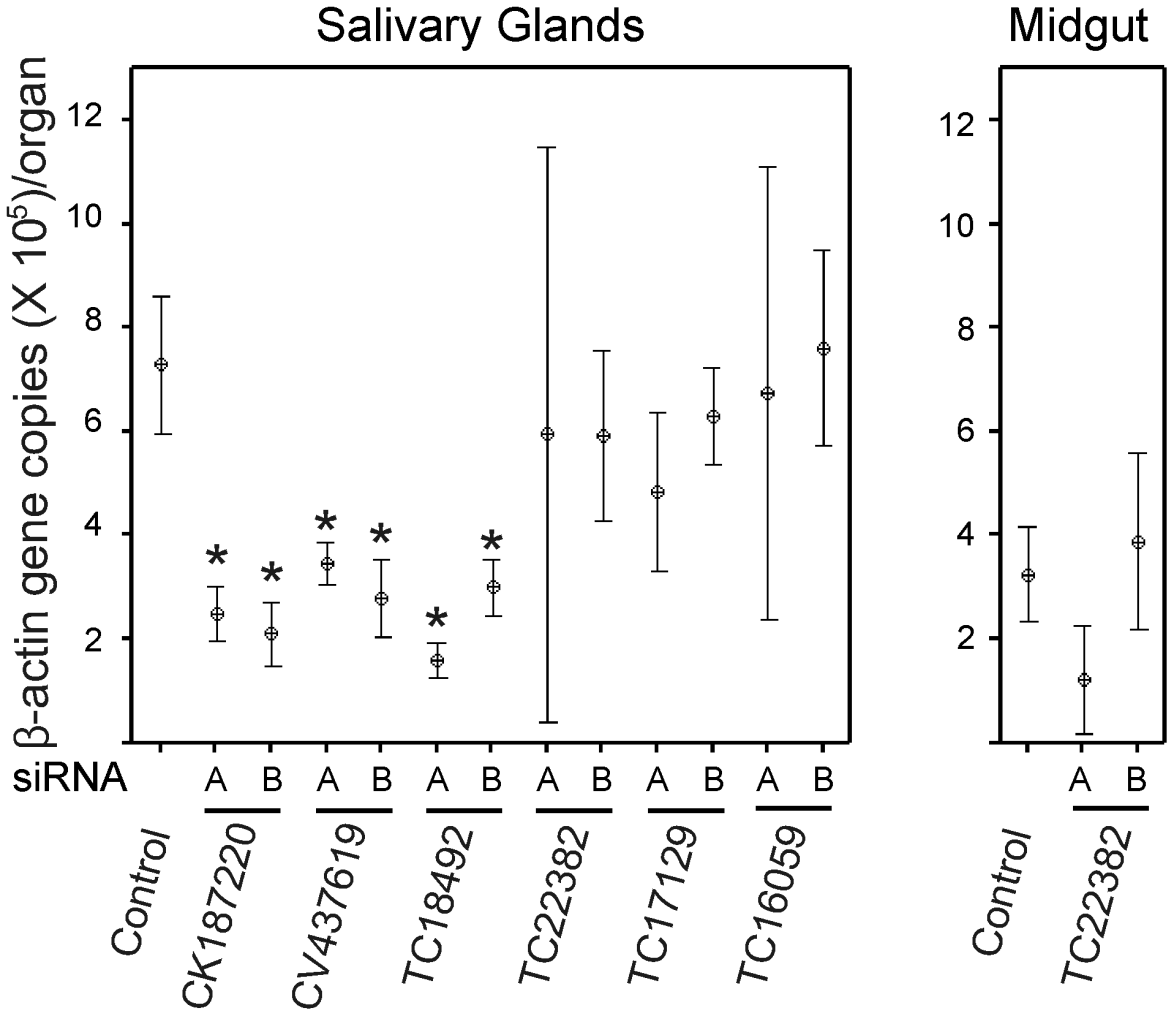
Effect of gene silencing on actin levels in tick organs. Interval plot of the distribution of *R. microplus* actin level in individual organs along the different gene-specific siRNA_A and siRNA_B injected groups, showing their central tendency and variability at a 95% confidence level. Asterisk (*) indicates statistically significant difference (p<0.05) when comparing to the respective control group.

It has been reported that gene silencing affected tick organ development generating smaller or altered tissues [15], [16], [17]. To investigate if silencing of our selected genes had an effect on the midgut or salivary gland, the tissue actin levels in individual organs were determined by qPCR for all ticks from all groups using aliquots from the same DNA samples used to detect and measure *A. marginale* infection. All samples showed detectable quantities of actin DNA (Figure 2). The amount of actin was statistically significantly lower (p<0.05) in salivary glands for groups injected with siRNAs for CK187220, CV437619, and TC18492. These groups also demonstrated lower *A. marginale* infection rates (Table 3). No statistically significant differences in actin levels were observed in midguts or salivary glands from groups injected with siRNAs corresponding to TC22382, TC17129 and TC16059, all of which had increased infection rates (Table 3). When comparing among control groups, actin quantity was significantly higher (p<0.05) in salivary glands than in midguts.

### Correlation between *A. marginale* Infection and Actin Levels

**Figure 3:**
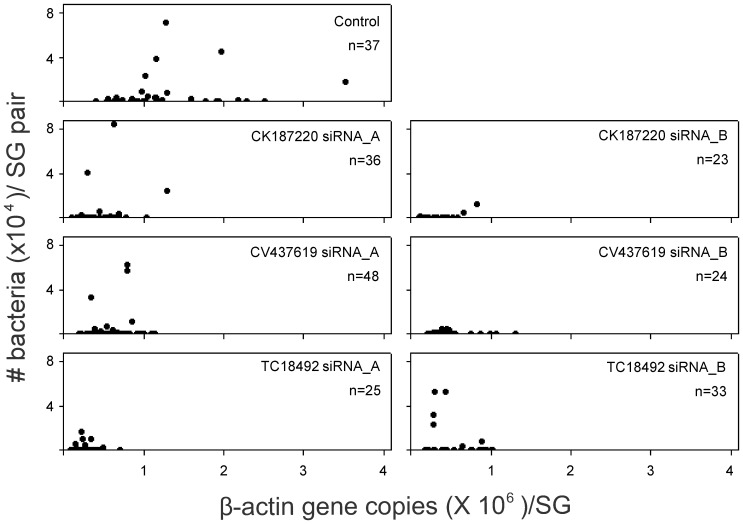
Correlation between *A. marginale* infection and actin levels. Scatterplot assessing the relationship between the two variables: *A. marginale* infection and actin levels, in individual pair of salivary glands from control group, and the two gene specific siRNA_A and siRNA_B injected groups for CK187220, CV437619, and TC18492. Both infected and uninfected tissues were included in the analysis; values from uninfected salivary glands can be visualized on the X-axis.

Salivary glands from control ticks had actin levels that ranged from 4.0×10^5^ to 3.5×10^6^. In contrast, the levels were consistently lower for three siRNA groups: CK187220, CV437619 and TC18492 (Figure 3). However, the actin level appeared to be independent of the infection level exhibited by the individual ticks in both the siRNA injected and control groups, with r values ranging from 0.05 to 0.69.

## Discussion

In the present study we tested two linked hypotheses. The first hypothesis, silencing of *R. microplus* genes significantly affects the *A. marginale* infection rate in the tick, was accepted based on the observation that gene silencing resulted in a decrease (CK187220, TC18492, CV437619) or an increase (TC22382, TC17129, TC16059) in the proportion of exposed ticks that acquired salivary gland infection (Table 3). Molecules that inhibited CK187220, TC18492, CV437619 expression or function would be candidates for development of transmission blocking therapeutics. Although there are no significant homologs to CK187220 in the current data bases, the nucleotide sequence of TC18492 showed 85% identity with three *Dermacentor variabilis* contigs (GenBank accession no. EZ525453.1, EZ524522.1, EZ533121.1) identified in pooled RNA isolated from unfed, uninfected adult male *D. variabilis*, partially fed males naturally infected with *A. marginale*, and unfed adult females injected with bacteria and fungi [18]. The deduced amino acid sequence of TC18492 also revealed 56% identity (E value 2×10^−67^) with an *Ixodes scapularis* putative secreted protein (GenBank accession no. XP_002435215.1). As TC18492 transcript and protein were, respectively, up-regulated in *A. marginale* and *Babesia bovis* infected *R. microplus* [5], [6], it raises the possibility that blocking TC18492 expression may be effective in blocking transmission of multiple pathogens.

The knockdown of CK187220, TC18492, and CV437619 gene expression following siRNA treatment was measured by normalization against actin. However, the level of salivary gland actin itself was decreased following injection with these siRNAs (Figure 2), thus the expression of these three genes was even more profoundly reduced than indicated by normalization against actin levels (Figure 1). This is especially relevant in the case of CV437619 where the silencing effect was not statistically significant when normalized against actin but there was a significant decrease in *A. marginale* infection rates. CV437619 shares 73% identity (E value, 8×10^−87^) with an *Ixodes scapularis* Tat binding protein 1 (TBP-1)-interacting protein (GenBank accession no. XP_002409139.1). The TBP-1 superfamily has several members that are components of the 26S proteasome, a basic multi-protein complex that degrades ubiquitinated proteins in an ATP-dependent fashion. That knockdown of all three genes, CK187220, TC18492, and CV437619, decreased overall actin levels suggests that the effects not only diminish pathogen infection but also affect normal cellular processes. Silencing of any one of the three genes did not completely abolish infection with *A. marginale*, however, suppression of gene combinations may have an additive or synergistic effect on blocking infection.

Silencing of TC22382, TC17129, and TC16059 resulted in increased *A. marginale* salivary gland infection rates. This suggests that the encoded proteins may normally function to inhibit infection, either directly or as members of cellular pathways. TC22382 has significant identity with NADH-ubiquinone reductases from ticks (*Amblyomma variegatum*, *Ixodes scapularis*), mosquitos (*Aedes aegypti, Anopheles gambiae*) and flies (*Drosophila pseudoobscura*). NADH-ubiquinone reductase is a conserved metabolic enzyme located in the inner mitochondrial membrane that catalyzes the transfer of electrons from NADH to coenzyme Q (CoQ). Interestingly, TC22382 is up regulated approximately three-fold in the midgut of adult male *Dermacentor andersoni* upon feeding [5], consistent with increased energy needs upon uptake of blood meal components, diuresis, and water balance. In addition, innate immune responses also require increased energy, thus decreased expression may lower innate responses in the midgut epithelium. Unfortunately, the 100% infection rate in the midgut of controls (Table 3), which may reflect a combination of residual ingested blood and colonization in the midgut, prevented determination if knockdown of TC22382 expression resulted in an increased midgut infection rate, as was observed in the salivary glands.

TC17129 had identity to a highly conserved region of glutamine synthetase from a wide range of organisms including: the red flour beetle *Tribolium castaneum* (GenBank accession no. XP_967731.1, 72% identity, E value 3×10^−133^), *Anopheles gambiae* (GenBank accession no. XP_312603.4, 71% identity, E value 1×10^−125^), and *Drosophila melanogaster* (GenBank accession no. ACV91641.1, NP_727525.1, NP_511123.2, 70% identity, E value 1×10^−122^). Glutamine synthetase plays an essential role in the metabolism of nitrogen by catalyzing the condensation of glutamate and ammonia to form glutamine, protecting the cell against excitotoxicity, or other adaptations that alleviate high levels of glutamate and ammonia [19]. In murine models of malaria and in *Schistosoma mansoni* infection of its molluscan host *Biomphalaria glabrata*, infection was associated with increased glutamine synthetase expression, suggested to be a protective mechanism against infection-induced increases in glutamate levels [20], [21]. The increased *A. marginale* infection rates upon TC17129 silencing in *R. microplus* ticks would be consistent with this role.

TC16059 has identity to aldehyde dehydrogenase from ticks including *Ixodes scapularis* and *Ambylomma variegatum*, as well several mosquito species, including *Culex quinquefasciatus*, *Aedes aegypti* and *Anopheles gambiae*. NAD(P)^+^-dependent enzymes in the aldehyde dehydrogenase superfamily are, in general, oxidoreductases that oxidize a wide range of endogenous and exogenous aliphatic and aromatic aldehydes, playing an important role in aldehyde detoxification. Additionally, they participate in 17 metabolic pathways such as glycolysis, gluconeogenesis, fatty acid and pyruvate metabolism, and pentose and glucuronate interconversions, and serve as binding proteins and osmoregulants. Aldehyde dehydrogenase is stress-induced and glucose-repressed, and has been shown to play a role in insecticide resistance in *Culex quinquefasciatus* [22]. TC16059 and other aldehyde dehydrogenases share a number of highly conserved residues necessary for catalysis and cofactor binding. TC16059 may have an infection-derived stress protective function against *A. marginale* infection, which would explain the increased infection rate (Table 3) associated with its silencing.

The second hypothesis, that silencing of the selected *R. microplus* genes affects the level of *A. marginale* within infected ticks, was rejected. This suggests that the targeted genes influence the pathogen at early steps in infection of the vector rather than in replication once infection is established. Importantly, the number of infected ticks has been shown to be a determinant of whether onward transmission to new mammalian hosts is successful, thus decreasing the infection rate, even if independent of the infection level, is likely to be successful in blocking transmission (Ueti et al., 2007, 2009; Agnes et al., 2010; Herndon et al, 2013).

Although there was variation in survival rates within and among treatment groups, these were not significantly different from the survival rate of the control group (Table 3). This is consistent with tick death being a consequence of the injection procedures rather than a specific effect of the siRNA. This interpretation is also supported by differential survival rates between tick cohorts injected with two different siRNAs targeting the same gene as a gene specific effect on tick survival would be expected to be similar between the siRNA_A and _B treatments. Nonetheless, the possibility of gene specific effects on tick growth and survival cannot be definitively excluded and can be addressed by including data such as engorgement weight to better assess growth and development. Refinement of the siRNA injection protocol to markedly improve survival in the control groups would facilitate detection of effects on tick growth and development and allow these to be discriminated from effects limited to pathogen entry and survival.

## Conclusions

Silencing of three *Rhipicephalus microplus* genes, CK187220, CV437619 and TC18492, significantly decreased the *Anaplasma marginale* infection rate in salivary glands, whereas gene silencing of TC22382, TC17129 and TC16059 significantly increased the infection rate in salivary glands. However in all cases of significant difference in the infection rate, the pathogen levels in the ticks that did become infected, were not significantly different. These results indicate that the targeted genes influence the pathogen at early steps in infection of the vector and provide specific targets for further testing that could lead to the development of small molecule inhibitors as transmission blocking chemotherapeutics.
